# Expression Levels of the ABCG2 Multidrug Transporter in Human Erythrocytes Correspond to Pharmacologically Relevant Genetic Variations

**DOI:** 10.1371/journal.pone.0048423

**Published:** 2012-11-15

**Authors:** Ildikó Kasza, György Várady, Hajnalka Andrikovics, Magdalena Koszarska, Attila Tordai, George L. Scheffer, Adrienn Németh, Gergely Szakács, Balázs Sarkadi

**Affiliations:** 1 Membrane Research Group of the Hungarian Academy of Sciences, Semmelweis University, Budapest, Hungary; 2 CellPharma Kft, Budapest, Hungary; 3 Hungarian National Blood Transfusion Service, Budapest, Hungary; 4 Free University Medical Center, Amsterdam, The Netherlands; 5 Institute of Molecular Pharmacology and Institute of Enzymology, Research Center for Natural Sciences, Hungarian Academy of Sciences (HAS), Budapest, Hungary; Indiana University School of Medicine, United States of America

## Abstract

We have developed a rapid, simple and reliable, antibody-based flow cytometry assay for the quantitative determination of membrane proteins in human erythrocytes. Our method reveals significant differences between the expression levels of the wild-type ABCG2 protein and the heterozygous Q141K polymorphic variant. Moreover, we find that nonsense mutations on one allele result in a 50% reduction in the erythrocyte expression of this protein. Since ABCG2 polymorphisms are known to modify essential pharmacokinetic parameters, uric acid metabolism and cancer drug resistance, a direct determination of the erythrocyte membrane ABCG2 protein expression may provide valuable information for assessing these conditions or for devising drug treatments. Our findings suggest that erythrocyte membrane protein levels may reflect genotype-dependent tissue expression patterns. Extension of this methodology to other disease-related or pharmacologically important membrane proteins may yield new protein biomarkers for personalized diagnostics.

## Introduction

Personalized medicine requires the development of biomarker diagnostic assays, reflecting individual variations and thus allowing tailored therapeutic interventions. Membrane proteins, contributing to about 30% of the total number of human proteins, play a key role in numerous human pathological conditions, while currently no simple assays are available for the determination of their tissue levels. Although genomic studies have established the pharmacological relevance of a large number of single nucleotide polymorphisms (SNP) and mutations, the direct correlation between genetic variations and membrane protein expression levels remains to be established. Clearly, as membrane proteins undergo complex processing, trafficking, and elimination, in many cases mRNA levels do not correspond to the ultimate protein expression in the relevant membrane.

Human erythrocytes express numerous integral membrane proteins (currently estimated at about 350 different proteins), including transporters, receptors, blood group antigens and proteins with confirmed involvement in human diseases [1], [2], [3], [4]. Although the expression of membrane proteins involved in erythropoiesis may not directly correspond to that observed in other specific tissues, the straightforward availability of blood samples and a simple and rapid, quantitative membrane protein assay platform could make the erythrocyte membrane widely applicable for biomarker analysis.

Based on this concept, we have developed an antibody-based quantitative assay for the determination of erythrocyte membrane proteins. As a pharmacologically relevant example, in this report we describe flow cytometry studies for measuring the expression of the ABCG2 multidrug transporter in human erythrocytes. The ABCG2 multidrug transporter is preferentially expressed in pharmacological barriers, in the liver, kidney and stem cells. This protein modulates the absorption, metabolism and toxicity of numerous drugs and xenobiotics, and causes multidrug resistance in cancer [5], [6], [7], [8], [9], [10], [11], [12]. Polymorphic variants or nonsense mutations of ABCG2 were found to be associated with interindividual variability in drug response to anticancer chemotherapy and the outcome of psoriasis or multiple sclerosis treatments [13], [14], [15], [16], [17], [18], [19], [20], [21], [22], [23]. Recently, a significant disease-association for a polymorphic *ABCG2* variant (resulting in ABCG2-Q141K) has been observed in gout [24], [25], [26], [27], [28].

It is well documented that mutations and polymorphisms of the *ABCG2* gene may cause mis-trafficking and early degradation that may contribute to decreased protein expression. A common variant of ABCG2 (c.421C>A; Q141K), with a variable allele frequency between 5–30% in various ethnic groups (see ref. [29]), was shown to decrease membrane protein expression in model cells, despite unchanged mRNA levels [30], [31], [32], [33], [34]. Still, a lower expression level of the ABCG2-Q141K variant has not been confirmed at physiologically relevant sites, given the difficulties in obtaining and processing human tissues.

It has been shown earlier that the erythrocyte membrane contains functional ABCG2 protein [35], [36], [37], [38]. Recently, two papers have been published, linking the rare blood group *Jun-* to the ABCG2 protein, showing that Jun- individuals have no ABCG2 expression in their red cell membranes. These individuals had mutations in their *ABCG2* gene on both alleles, resulting in early termination of transcription, while had no apparent disease conditions [39], [40].

In this report we show that individuals heterozygous for the potentially miss-processed ABCG2 variant (Q141K) have significantly lower ABCG2 protein expression in their red cells than individuals carrying the wild-type *ABCG2* gene. Moreover, heterozygous individuals with an *ABCG2* nonsense mutation on one allele, have about 50% reduction in their red cell ABCG2 protein expression. These data suggest that determination of the ABCG2 protein expression in the erythrocyte membrane may provide clinically valuable information for assessing the role of this protein in relevant diseases, metabolic conditions, or the efficiency and/or toxicity of drug treatments.

## Methods

Anticoagulated blood samples of healthy volunteers (47 unrelated individuals and 14 family members of two probands selected from the donor cohort) were fixed in paraformaldehyde (PFA), stained with monoclonal antibodies specifically recognizing membrane proteins, and subjected to flow cytometry (FACS). In parallel, genomic DNA was isolated from the blood samples; common SNPs of the *ABCG2* gene were screened by LightCycler allelic discrimination system, while mutations were determined by direct sequencing. All subjects gave their written informed consent to participate in the study. This study was approved by the regional ethical committees, and all procedures were performed in accordance with the Declaration of Helsinki.

### Flow Cytometry

Freshly drawn human blood (25 µl) was diluted in 4 ml of phosphate buffered saline (PBS) containing 1% PFA and fixed for 60 min at 25°C. In preliminary experiments we have analyzed the effects of various PFA concentrations (0.5–4%), phosphate or Tris buffer concentrations, fixation periods (10–120 min) and fixation temperatures (4–37°C), and found the above conditions allowing optimum formation of mixed intact red cell/ghost populations as well as antibody recognition.

After fixation the cells were centrifuged at 3,000 × g for 10 min and the pellet was resuspended in 100 µl PBS. Antibody staining was performed for 40 min at 37°C by using the BXP34 (final concentration 5 µg/ml) and the BXP21 (7 µg/ml) generated by G. Scheffer [41]), the 5D3 (final concentration 5 µg/ml, BD-Pharmingen 562167) monoclonal antibodies (mAb) specific for ABCG2, or the respective IgG (immunoglobulin G) control antibodies (IgG1: Invitrogen, MG100, IgG2a: Invitrogen, MG2A00, IgG2b: Invitrogen, MG2B00, final concentrations 5 µg/ml). It should be noted that BXP21 can also be used for Western blotting, while the BXP34 mAb does not recognize ABCG2 on Western blots but specifically interacts with the protein in tissue preparations [42]. Antibody concentrations for ABCG2 labeling in red cells were carefully calibrated to provide maximum labeling in a full range of protein expression (see below).

For staining the red cell membrane plasma membrane calcium pump (PMCA) protein we used the 5F10 monoclonal antibody (final concentration 4 µg/ml, ABCAM, Ab2825), recognizing all 4 human PMCA isoforms having common peptide epitopes [43]. As shown in the Supplementary materials for the 5F10 monoclonal antibody, a calibration of a wide range of the applied antibody assured maximum labeling for the red cell protein.

After washing out the primary antibodies, secondary antibodies corresponding to the IgG type and labeled with phycoerythrin (PE) were added to the cells (goat anti-mouse (GAM) IgG1-PE: Invitrogen P21129, GAM IgG2a-PE: Invitrogen P21139, GAM IgG2b-PE: Invitrogen, P21149, final concentrations 5 µg/ml), incubated for 30 min at 37°C, washed and resuspended in PBS. In parallel tubes cells were directly stained with FITC-conjugated anti-Glycophorin A (GlyA) monoclonal antibody (final concentration 5 µg/ml, Gly-A-FITC: Beckman Coulter, 2212). We have used several batches of the respective antibodies that all showed similar results at the described concentrations.

**Figure 1:**
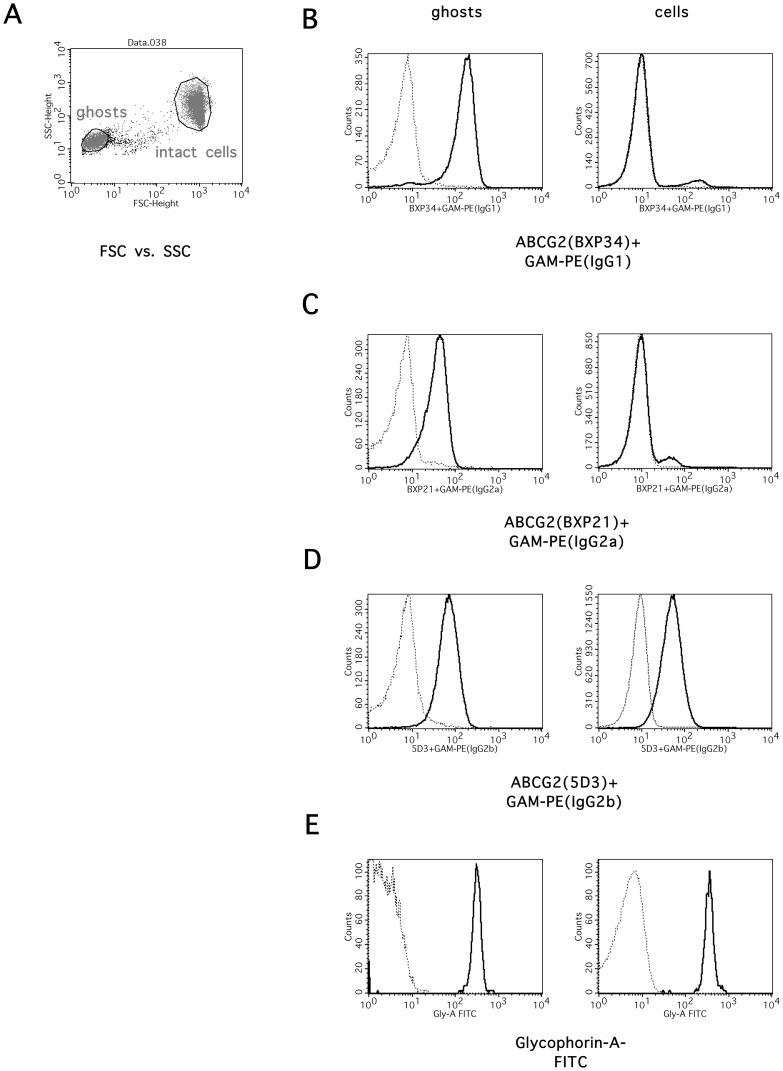
Quantitative determination of ABCG2 expression in the erythrocyte membrane by flow cytometry. Anticoagulated blood samples of healthy volunteers were fixed in paraformaldehyde, stained with monoclonal antibodies recognizing human ABCG2, and subjected to flow cytometry (see Online Methods). Antibody staining was performed by BXP34 (Panel B), BXP21 (Panel C) and 5D3 (Panel D) mAbs specific for ABCG2, or the respective IgG control antibodies, followed by staining with PE-labeled secondary antibodies. In Panel E cells were stained with a FITC-conjugated anti-Glycophorin A mAb. Intact erythrocytes and erythrocyte ghost were gated based on the forward scatter (FSC) and side scatter (SSC) parameters (Panel A).

Intact red cells and erythrocyte ghost were gated based on the forward scatter (FSC) and side scatter (SSC) parameters. Both fractions were analyzed for antibody staining by a FACSCalibur flow cytometer (excitation wavelength: 488 nm (Argon ion laser) emission filters: 585/42 nm for PE, 530/30 for FITC – for details see Fig. 1). A careful optimization for maximum antibody labeling was carried out for all monoclonal antibodies (see Results). For ABCG2 expression controls, we used K562 cells retrovirally transduced to express ABCG2, as described previously [44]. As described earlier [44], PFA fixation of the membrane ABCG2 protein eliminates the conformation-sensitivity of its interaction with the 5D3 antibody, and provides maximum labeling.

### Determination of Expression Levels

**Figure 2:**
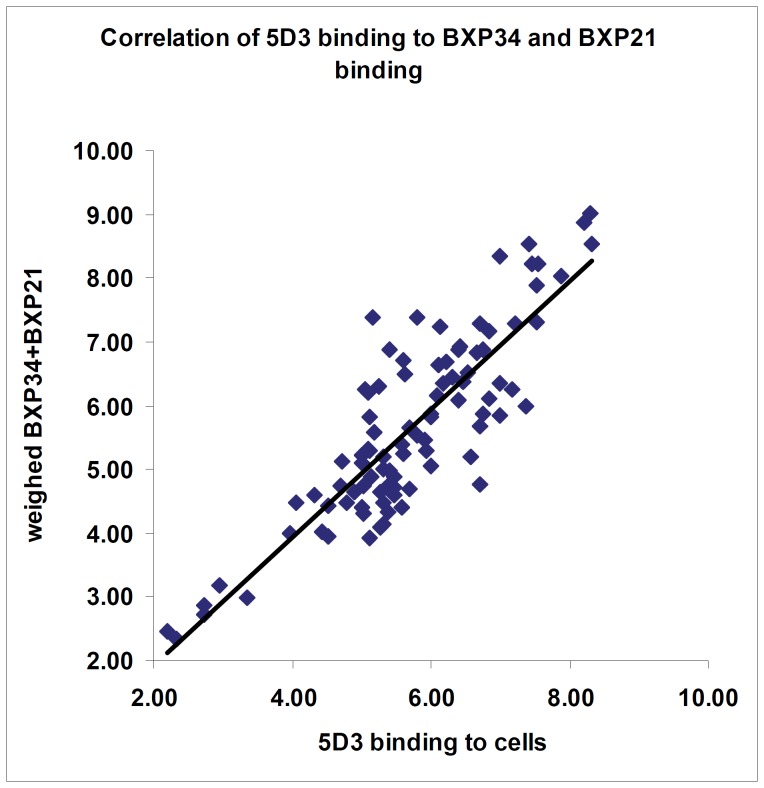
Detection of ABCG2 expression level by specific monoclonal antibodies. The Figure shows the correlation of the ABCG2 expression level detected by the 5D3 monoclonal antibody in the fixed whole cells, recognizing the ABCG2 protein on an extracellular epitope, and the weighed average of BXP21 and BXP34 antibodies (BXP34/3+BXP21)/2, recognizing intracellular epitopes of ABCG2. The correlation is linear; the value of the correlation coefficient R is 0.859.

In order to use a combination of all relevant anti-ABCG2 antibodies for quantifying ABCG2 expression in the red cell membranes, we calculated an average binding factor, based on the relative staining efficiencies observed. In all experiments BXP34 gave 3 times greater relative staining (as measured by the Geo Mean values of the gated populations) than either the BXP21 or the 5D3 antibodies (the exact epitopes of the mAbs are unknown), therefore in the calculated ABCG2 factor we used the following equation: ((BXP34/3)+BXP21+5D3)/3. As documented in Figure 2, a linear correlation between the calculated average of BXP34 and BXP21 binding ((BXP34/3)+BXP21)/2) in the red cell ghosts and the values measured for the cell-surface reactive, human-specific 5D3 binding in whole red cells was observed. The calculated intra- and inter assay variations are presented in the Results section and in the Supplementary Materials.

### Additional Methods

In order to compare the relative expression of ABCG2 in the red cell membrane to those in known expression systems, we performed Western blot experiments using isolated red cell membranes, isolated Sf9 insect cell membranes expressing the human ABCG2 protein, and A431 cells overexpressing ABCG2 (see Supplementary Materials and refs. [45], [46], [47]. In accordance with previous data in the literature [35], [36], [37], [38], [39], we detected both the monomeric and dimeric forms of ABCG2 in the red cell membrane but found that this assay is not suitable for the proper quantitation of small changes in ABCG2 expression (see Supplementary Materials).

The ABCG2 transport function in inside-out red cell membrane vesicles has been determined earlier [35], [36]. We found that in intact human erythrocyte this function could not be properly studied by flow cytometry, as the fluorescence of all the available ABCG2-transported compounds was strongly quenched by the high concentrations of hemoglobin.

### Genetic Analysis

For *ABCG2* polymorphism and mutation studies genomic DNA was isolated from EDTA anticoagulated peripheral blood by the Gentra Purege Blood Kit (Qiagen, Hamburg, Germany). The most common SNPs in ABCG2 [V12M (c.34G>A, p.12Val>Met in exon 2, SNP database ID: rs2231137) and Q141K (c.421C>A, p.141Gln>Lys in exon 5, SNP database ID: rs2231142] were genotyped using the LightCycler480 (Roche Diagnostics, Basle, Switzerland) allelic discrimination system as described previously in detail [45]. Sanger sequencing of the *ABCG2* coding region and exon-intron boundaries (exons 2–16) was performed by the Applied Biosystems 310 Genetic Analyzer (Life Technologies, Carlsbad, USA) [40].

## Results and Discussion

We have used a flow cytometry based assay for the quantitative determination of ABCG2 expression in erythrocytes. We found that the forward/side scatter plot delineates two major populations, corresponding to PFA-fixed intact erythrocytes and erythrocyte membrane “ghosts”, respectively (Fig. 1A). Antibodies recognizing intracellular epitopes of ABCG2, that is BXP21 and BXP34 (see refs. [41], [42]), bind to ghosts that are accessible from both sides of the membrane (Fig. 1B-C), but not to the fixed, intact erythrocytes. Conversely, the 5D3 monoclonal antibody, recognizing an extracellular epitope of the ABCG2 protein (see ref. [46]) shows binding to both PFA-fixed whole red cells and the ghost fraction (Fig. 1D). The different membrane accessibility in the two erythrocyte fractions was also confirmed using antibodies recognizing an extracellular Glycophorin A epitope (Fig. 1E), or the intracellular epitopes of the human plasma membrane calcium ATPase protein ([43], plasma membrane calcium ATPase, PMCA, see below). Moreover, retention of the viability dye, calcein was observed in the fixed whole cell fraction, while not in the ghost fraction (data not shown).

As documented in Fig. 1, all three ABCG2-specific antibodies detected significant expression of ABCG2 in erythrocytes. In order to allow quantitative protein determination, we titrated the antibodies to obtain maximum binding. After antibody titration all the relevant monoclonal antibodies were applied in concentrations exceeding maximum binding levels (for the PMCA protein see the Supplementary Materials). Since the exact epitopes of the ABCG2 mAbs are unknown, and BXP34 labeling consistently gave three times greater relative staining than either the BXP21 or the 5D3 antibodies, for ABCG2 expression we used a weighed average, named “RBC-G2 factor” (see Methods). As shown in Figure 2, a linear correlation between the weighed average binding of the two mAbs, BXP34 and BXP21, recognizing intracellular epitopes in the ghost fraction, and the binding of the cell-surface reactive 5D3 mAb (in whole red cells) was observed.

We have performed detailed intra-assay and interassay analyses which gave acceptable reproducibility. The performance of the assays with different antibody stainings is shown in the Supplementary Materials, Table S1. The intraassay imprecision (coefficient of variation [CV%] expressed as SD/mean %) for the ABCG2 factor was 5.3%, as measured from 8 peripheral blood aliquots from the same healthy volunteer on the same day. Interassay imprecision was calculated from samples taken from the same individual on different days. We obtained an interassay CV% for ABCG2 factor of 10.1%, when using results obtained from 29 individuals with replicate measurements (using 2–4 replicates in each case).

In additional control experiments we used intact or lysed rat and pig red cells to analyze the specificity of the anti-ABCG2 antibodies applied. We found that the three human-specific anti-ABCG2 antibodies and the anti-Glycophorin A monoclonal antibody did not label rat or pig red cells.

**Figure 3:**
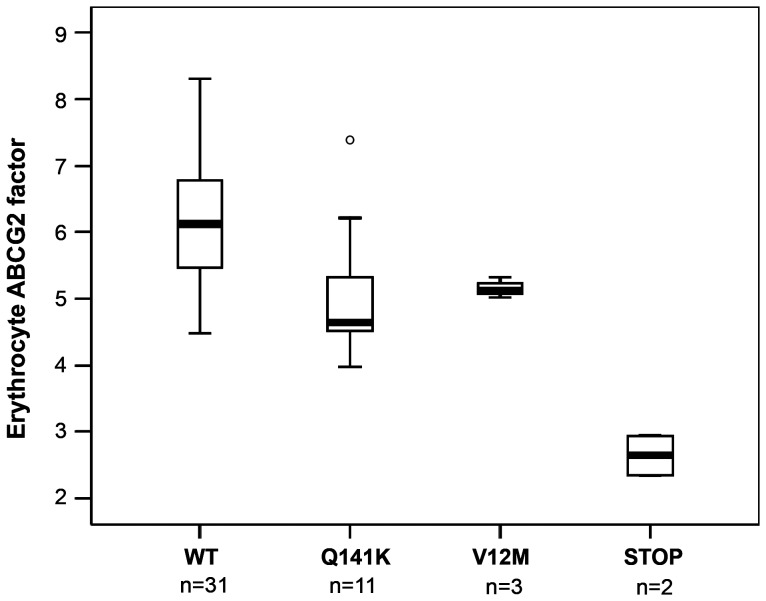
ABCG2 is differentially expressed in the red blood cells of individuals carrying homozygous wild-type, heterozygous polymorphic or premature stop codon mutant ABCG2 alleles. Boxplot presentation showing the median and the 25–75^th^ percentiles, whiskers represent 10–90^th^ percentiles. ABCG2 expression is calculated based on the combined reactivity of anti-ABCG2 mAbs (RBC-G2 factor – see Methods). Labels: individuals carrying wild-type ABCG2 (WT), polymorphic (Q141K, V12M) ABCG2 alleles, or a heterozygous stop mutation (STOP).

Next, we examined if the red cell membrane ABCG2 protein levels correlated with pharmacologically relevant polymorphisms that are known to influence protein expression in model cells. For this purpose we quantified the expression of the erythrocyte ABCG2 in 47 unrelated, healthy individuals that were also screened for the presence of two most prevalent ABCG2 polymorphic variants found in the Caucasian population (V12M and Q141K) [29]. Significant ABCG2 levels, encompassing a wide range of expression were detected in the red blood cells of all individuals. Differences of the erythrocyte ABCG2 expression could not be attributed to age or sex. However, when the samples were grouped according to their genotypes, we found that the red blood cells of individuals carrying the heterozygous Q141K variant exhibited significantly lower expression of ABCG2 (5.27±1.19), as compared to homozygous wild-type individuals (6.13±0.61, p = 0.011) (Fig. 3). There was no significant difference between homozygous wild-type individuals and heterozygous V12M carriers, although the number of the carriers of this variant was relatively low. As a summary of the *ABCG2* polymorphism analysis data, among the 47 donors we found 11 individuals with the heterozygous presence of the DNA sequence coding for the Q141K variant (carrier frequency: 23.4%, allele frequency: 11.7±6.6%), and 3 individuals with the heterozygous presence of the V12M variant (carrier frequency: 6.4%; allele frequency: 3.2±3.6%). These results correspond to the general polymorphism distributions in the European populations (see [29]).

Interestingly, we found two unrelated individuals showing much lower than the average (about 50%) erythrocyte ABCG2 expression (2.65±0.29) (Fig. 3). Sequencing of the entire coding region of the *ABCG2* gene revealed that these individuals carry heterozygous mutations resulting in premature termination (without further polymorphic variations). A nonsense mutation, causing an arginine to stop codon change at codon 236 in exon 7 (c.706C>T, p.R236X, rs140207606 described previously [39], [40]) was found in heterozygous form in proband 1. A small deletion (c.791_792delTT, L264HfsX14 described in a recent paper by [39]) causing frameshift and the truncation of the protein was found in proband 2.

**Figure 4:**
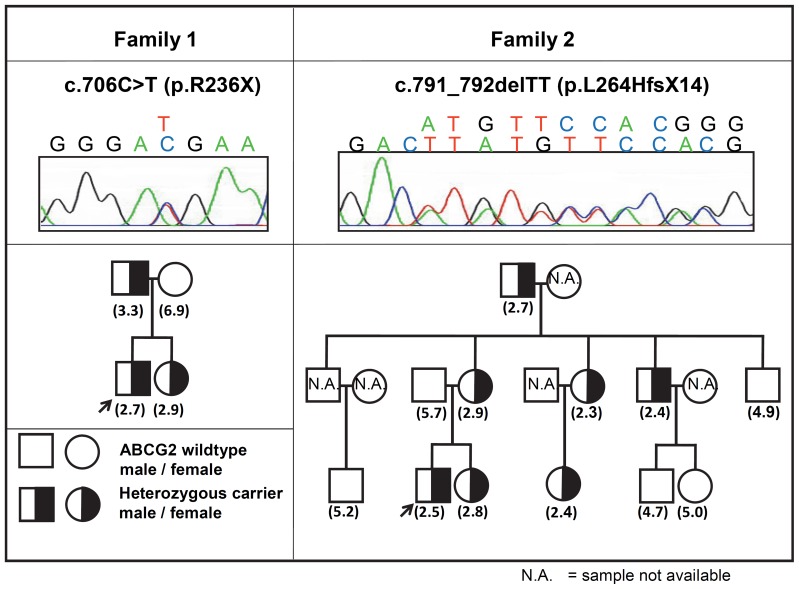
Pedigrees of two families carrying different ABCG2 premature stop mutations – co-segregation of the heterozygous mutation with reduced erythrocyte ABCG2 expression levels. Blood samples obtained from the 14 family members of the two healthy volunteer probands, carrying the premature stop mutations (see Fig. 3 -indicated with arrowheads) were analyzed for ABCG2 expression and the respective mutations. The RBC-G2 factor values, reflecting ABCG2 expression in erythrocytes, are shown in parentheses. Family members not available for blood donation are labeled by N.A.

In order to clarify if a direct relationship exists between the heterozygous stop mutations and the erythrocyte ABCG2 expression levels, we obtained blood samples from the family members of the two probands carrying these premature termination mutations. As shown in Fig. 4, we found a co-segregation of the reduced erythrocyte ABCG2 expression levels (about 50% reduction) and the respective mutations in the two families. These findings show a direct correlation between ABCG2 variants and erythrocyte membrane expression, and indicate a general bi-allelic expression pattern for ABCG2, as has been suggested, based on mRNA data [47], [48], [49].

**Figure 5:**
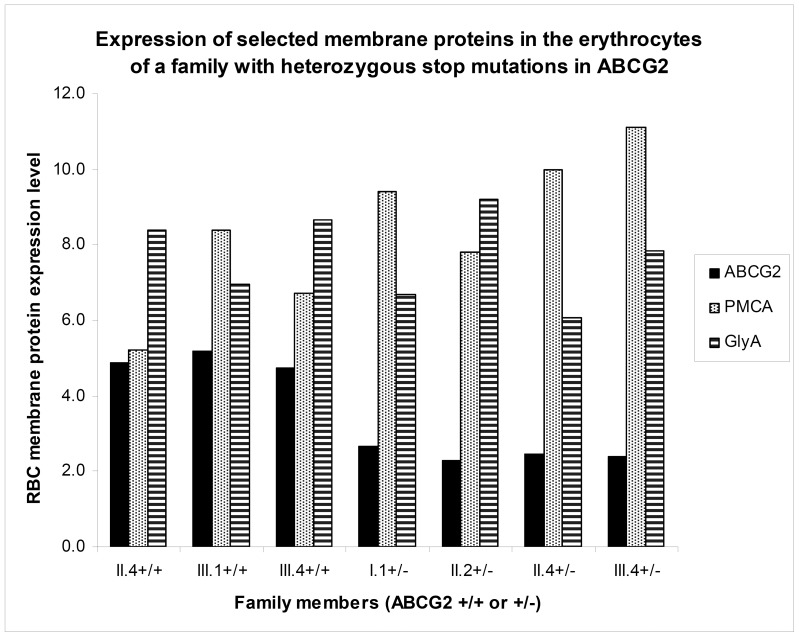
Expression of three selected membrane proteins in the erythrocyte membrane in family 2 with members having a heterozygous frameshift mutation on one of the *ABCG2* alleles (labeled as+/−), as compared to ABCG2 wild type individuals of the same family (labeled as+/+). Family members are labeled according to generations (I, II, III), and blood samples obtained (1–4).

In order to examine the specificity of the lower ABCG2 expression related to the genotype changes, we have also analyzed the relative quantitative expression of other erythrocyte membrane proteins. Here we document the compared quantitative expression patterns of the calcium pump protein, PMCA, the Glycophorin A protein, and the ABCG2 protein within a family, in which we found individuals with low ABCG2 expression, due to premature termination of ABCG2 transcription on one allele (Figure 5). The 5F10 monoclonal antibody applied here specifically recognizes all four PMCA isoforms, containing a common epitope [43]. As shown, while the pattern of ABCG2 expression showed significant differences corresponding to the presence of a heterozygous mutation (labeled as+/−), PMCA or GlyA expression levels, although with some variations, were independent from the ABCG2 expression levels.

As a summary, we have developed a simple and reliable flow cytometry assay to quantitate the expression of the human ABCG2 protein in erythrocytes, and found a close correlation between protein expression and the ABCG2 genotype. This technology has major advantages as compared to other available methods.

As documented in the Supplementary materials, we have performed detailed Western blotting studies of the ABCG2 in the isolated membranes or the whole red cells of donors with variable expression levels. However, although the ABCG2 bands in the red cells can be well detected, this laborious and time consuming technology cannot be properly used to evaluate quantitative differences in the ABCG2 expression levels between various blood samples.

Another possible strategy for the absolute quantification of a membrane protein like ABCG2 is to use quantitative LC-MS/MS technology, e.g. described in reference [50]. However, the LC-MS/MS measurements require highly specialized, expensive equipment and detailed standardization of the protein fragmentation, internal standards, etc. This was not the goal of the present work, as the relative red cell membrane expression levels were correlated with the respective genetic backgrounds. We suggest that the flow-cytometry method presented here is more suitable for a rapid, widely applicable clinical laboratory diagnostics.

In this study we show that the erythrocyte ABCG2 levels correspond to the genetic background, thus may reflect the overall tissue expression patterns of this protein. We suggest that the method presented here may provide the basis for the development of generally applicable membrane protein biomarkers. It has been documented that ABCG2 expression is regulated by various xenobiotics, drugs, or stress conditions, e.g. hypoxia – see [10], [11], [12], [29]. In a larger cohort of donors and hematological patients we are currently studying the role of drug treatment and environmental factors in the erythrocyte expression levels of the ABCG2 protein. These may provide clinically valuable data in the context of drug absorption, distribution, and toxicity, as well as predicting treatment efficiency and potential multidrug resistance. A quantitative measure of the expression of ABCG2, shaping ADME-Tox properties and drug sensitivity, should significantly promote a personalized approach in pharmacology. Although the ABCG2 expression in the cancerous tissues does not uniquely depend on the overall genetic background (special regulatory changes or gene rearrangements may occur), the red cell ABCG2 protein levels may reflect the physiological tissue expression patterns, which modify the efficiency or toxicity of the cancer drug treatment. These questions are still to be explored in larger scale studies.

In addition, the above simple methodology may be applicable for a wide variety of clinically relevant membrane proteins, expressed in the erythrocytes. As shown in Figure 5 and in the Supplementary Materials, the plasma membrane calcium pump (PMCA) or Glycophorin A can be quantitatively detected in the human red cells by this method. In our current experiments, not presented here in detail, we have found that the above described technology can be applied to quantitatively assess the expression of several other membrane proteins (including ABCA1, ABCB6, ABCC1, ABCC3, and ABCC4). We are currently performing population studies to evaluate the significance of SNPs and mutations in the expression levels of these membrane proteins.

## Supporting Information

Figure S1 **Western blot analysis of isolated red cell membrane preparations, compared to ABCG2-expressing Sf9 cell membrane preparations or A431 tumor cells, expressing ABCG2** [**1**] **.**
(TIF)

Figure S2 **Comparison of ABCG2 expression on Western blot – detection by BXP21 antibody.**
(TIF)

Figure S3 **Calibration of 5F10 antibody binding and saturation.**
(TIF)

Materials S1 (DOC)

## References

[1] Alexandre BM (2010) Proteomic mining of the red blood cell: focus on the membrane proteome. Expert Rev Proteomics 7: 165–168. 20377381 10.1586/epr.09.96

[2] Goodman SR, Kurdia A, Ammann L, Kakhniashvili D, Daescu O (2007) The human red blood cell proteome and interactome. Exp Biol Med (Maywood) 232: 1391–1408. 18040063 10.3181/0706-MR-156

[3] Pasini EM, Kirkegaard M, Mortensen P, Lutz HU, Thomas AW, et al (2006) In-depth analysis of the membrane and cytosolic proteome of red blood cells. Blood 108: 791–801. 16861337 10.1182/blood-2005-11-007799

[4] Pasini EM, Lutz HU, Mann M, Thomas AW (2010) Red blood cell (RBC) membrane proteomics–Part I: Proteomics and RBC physiology. J Proteomics 73: 403–420. 19540949 10.1016/j.jprot.2009.06.005

[5] Allen JD, Schinkel AH (2002) Multidrug resistance and pharmacological protection mediated by the breast cancer resistance protein (BCRP/ABCG2). Mol Cancer Ther 1: 427–434. 12477055

[6] Doyle LA, Yang W, Abruzzo LV, Krogmann T, Gao Y, et al (1998) A multidrug resistance transporter from human MCF-7 breast cancer cells. Proc Natl Acad Sci U S A 95: 15665–15670. 9861027 10.1073/pnas.95.26.15665 PMC28101

[7] Jonker JW, Buitelaar M, Wagenaar E, Van Der Valk MA, Scheffer GL, et al (2002) The breast cancer resistance protein protects against a major chlorophyll-derived dietary phototoxin and protoporphyria. Proc Natl Acad Sci U S A 99: 15649–15654. 12429862 10.1073/pnas.202607599 PMC137771

[8] Krishnamurthy P, Ross DD, Nakanishi T, Bailey-Dell K, Zhou S, et al (2004) The stem cell marker Bcrp/ABCG2 enhances hypoxic cell survival through interactions with heme. J Biol Chem 279: 24218–24225. 15044468 10.1074/jbc.M313599200

[9] Krishnamurthy P, Schuetz JD (2006) Role of ABCG2/BCRP in biology and medicine. Annu Rev Pharmacol Toxicol 46: 381–410. 16402910 10.1146/annurev.pharmtox.46.120604.141238

[10] Robey RW, Polgar O, Deeken J, To KW, Bates SE (2007) ABCG2: determining its relevance in clinical drug resistance. Cancer Metastasis Rev 26: 39–57. 17323127 10.1007/s10555-007-9042-6

[11] Sarkadi B, Homolya L, Szakacs G, Varadi A (2006) Human multidrug resistance ABCB and ABCG transporters: participation in a chemoimmunity defense system. Physiol Rev 86: 1179–1236. 17015488 10.1152/physrev.00037.2005

[12] Szakacs G, Varadi A, Ozvegy-Laczka C, Sarkadi B (2008) The role of ABC transporters in drug absorption, distribution, metabolism, excretion and toxicity (ADME-Tox). Drug Discov Today 13: 379–393. 18468555 10.1016/j.drudis.2007.12.010

[13] Akasaka K, Kaburagi T, Yasuda S, Ohmori K, Abe K, et al (2010) Impact of functional ABCG2 polymorphisms on the adverse effects of gefitinib in Japanese patients with non-small-cell lung cancer. Cancer Chemother Pharmacol 66: 691–698. 20035425 10.1007/s00280-009-1211-6

[14] Cotte S, von Ahsen N, Kruse N, Huber B, Winkelmann A, et al (2009) ABC-transporter gene-polymorphisms are potential pharmacogenetic markers for mitoxantrone response in multiple sclerosis. Brain 132: 2517–2530. 19605531 10.1093/brain/awp164

[15] Cusatis G, Gregorc V, Li J, Spreafico A, Ingersoll RG, et al (2006) Pharmacogenetics of ABCG2 and adverse reactions to gefitinib. J Natl Cancer Inst 98: 1739–1742. 17148776 10.1093/jnci/djj469

[16] Gardner ER, Burger H, van Schaik RH, van Oosterom AT, de Bruijn EA, et al (2006) Association of enzyme and transporter genotypes with the pharmacokinetics of imatinib. Clin Pharmacol Ther 80: 192–201. 16890580 10.1016/j.clpt.2006.05.003

[17] Honjo Y, Morisaki K, Huff LM, Robey RW, Hung J, et al (2002) Single-nucleotide polymorphism (SNP) analysis in the ABC half-transporter ABCG2 (MXR/BCRP/ABCP1). Cancer Biol Ther 1: 696–702. 12642696 10.4161/cbt.322

[18] Imai Y, Nakane M, Kage K, Tsukahara S, Ishikawa E, et al (2002) C421A polymorphism in the human breast cancer resistance protein gene is associated with low expression of Q141K protein and low-level drug resistance. Mol Cancer Ther 1: 611–616. 12479221

[19] Ishikawa T, Nakagawa H, Hagiya Y, Nonoguchi N, Miyatake S, et al (2010) Key Role of Human ABC Transporter ABCG2 in Photodynamic Therapy and Photodynamic Diagnosis. Adv Pharmacol Sci 2010: 587306. 21188243 10.1155/2010/587306 PMC3003952

[20] Li J, Cusatis G, Brahmer J, Sparreboom A, Robey RW, et al (2007) Association of variant ABCG2 and the pharmacokinetics of epidermal growth factor receptor tyrosine kinase inhibitors in cancer patients. Cancer Biol Ther 6: 432–438. 17312388 10.4161/cbt.6.3.3763

[21] Sparreboom A, Gelderblom H, Marsh S, Ahluwalia R, Obach R, et al (2004) Diflomotecan pharmacokinetics in relation to ABCG2 421C>A genotype. Clin Pharmacol Ther 76: 38–44. 15229462 10.1016/j.clpt.2004.03.003

[22] Tamura A, Onishi Y, An R, Koshiba S, Wakabayashi K, et al (2007) In vitro evaluation of photosensitivity risk related to genetic polymorphisms of human ABC transporter ABCG2 and inhibition by drugs. Drug Metab Pharmacokinet 22: 428–440. 18159130 10.2133/dmpk.22.428

[23] Warren RB, Smith RL, Campalani E, Eyre S, Smith CH, et al (2008) Genetic variation in efflux transporters influences outcome to methotrexate therapy in patients with psoriasis. J Invest Dermatol 128: 1925–1929. 18256692 10.1038/jid.2008.16

[24] Basseville A, Bates SE (2011) Gout, genetics and ABC transporters. F1000 Biol Rep 3: 23. 22065982 10.3410/B3-23 PMC3206739

[25] Ichida K, Matsuo H, Takada T, Nakayama A, Murakami K, et al (2012) Decreased extra-renal urate excretion is a common cause of hyperuricemia. Nat Commun 3: 764. 22473008 10.1038/ncomms1756 PMC3337984

[26] Matsuo H, Takada T, Ichida K, Nakamura T, Nakayama A, et al (2009) Common defects of ABCG2, a high-capacity urate exporter, cause gout: a function-based genetic analysis in a Japanese population. Sci Transl Med 1: 5ra11. 10.1126/scitranslmed.3000237 20368174

[27] Robey RW, Ierano C, Zhan Z, Bates SE (2011) The challenge of exploiting ABCG2 in the clinic. Curr Pharm Biotechnol 12: 595–608. 21118093 10.2174/138920111795163913 PMC3091815

[28] Yang Q, Kottgen A, Dehghan A, Smith AV, Glazer NL, et al (2010) Multiple genetic loci influence serum urate levels and their relationship with gout and cardiovascular disease risk factors. Circ Cardiovasc Genet 3: 523–530. 20884846 10.1161/CIRCGENETICS.109.934455 PMC3371395

[29] Cervenak J, Andrikovics H, Ozvegy-Laczka C, Tordai A, Nemet K, et al (2006) The role of the human ABCG2 multidrug transporter and its variants in cancer therapy and toxicology. Cancer Lett 234: 62–72. 16337740 10.1016/j.canlet.2005.01.061

[30] Furukawa T, Wakabayashi K, Tamura A, Nakagawa H, Morishima Y, et al (2009) Major SNP (Q141K) variant of human ABC transporter ABCG2 undergoes lysosomal and proteasomal degradations. Pharm Res 26: 469–479. 18958403 10.1007/s11095-008-9752-7 PMC2628956

[31] Kondo C, Suzuki H, Itoda M, Ozawa S, Sawada J, et al (2004) Functional analysis of SNPs variants of BCRP/ABCG2. Pharm Res 21: 1895–1903. 15553238 10.1023/b:pham.0000045245.21637.d4

[32] Mizuarai S, Aozasa N, Kotani H (2004) Single nucleotide polymorphisms result in impaired membrane localization and reduced atpase activity in multidrug transporter ABCG2. Int J Cancer 109: 238–246. 14750175 10.1002/ijc.11669

[33] Morisaki K, Robey RW, Ozvegy-Laczka C, Honjo Y, Polgar O, et al (2005) Single nucleotide polymorphisms modify the transporter activity of ABCG2. Cancer Chemother Pharmacol 56: 161–172. 15838659 10.1007/s00280-004-0931-x

[34] Nakagawa H, Toyoda Y, Wakabayashi-Nakao K, Tamaki H, Osumi M, et al (2011) Ubiquitin-mediated proteasomal degradation of ABC transporters: a new aspect of genetic polymorphisms and clinical impacts. J Pharm Sci 100: 3602–3619. 21567408 10.1002/jps.22615

[35] de Wolf CJ, Yamaguchi H, van der Heijden I, Wielinga PR, Hundscheid SL, et al (2007) cGMP transport by vesicles from human and mouse erythrocytes. Febs J 274: 439–450. 17229149 10.1111/j.1742-4658.2006.05591.x

[36] Leimanis ML, Georges E (2007) ABCG2 membrane transporter in mature human erythrocytes is exclusively homodimer. Biochem Biophys Res Commun 354: 345–350. 17250810 10.1016/j.bbrc.2006.12.219

[37] Maliepaard M, Scheffer GL, Faneyte IF, van Gastelen MA, Pijnenborg AC, et al (2001) Subcellular localization and distribution of the breast cancer resistance protein transporter in normal human tissues. Cancer Res 61: 3458–3464. 11309308

[38] Zhou S, Zong Y, Ney PA, Nair G, Stewart CF, et al (2005) Increased expression of the Abcg2 transporter during erythroid maturation plays a role in decreasing cellular protoporphyrin IX levels. Blood 105: 2571–2576. 15546952 10.1182/blood-2004-04-1566 PMC4757428

[39] Saison C, Helias V, Ballif BA, Peyrard T, Puy H, et al (2012) Null alleles of ABCG2 encoding the breast cancer resistance protein define the new blood group system Junior. Nat Genet 44: 174–177. 22246505 10.1038/ng.1070 PMC3653631

[40] Zelinski T, Coghlan G, Liu XQ, Reid ME (2012) ABCG2 null alleles define the Jr(a-) blood group phenotype. Nat Genet 44: 131–132. 22246507 10.1038/ng.1075

[41] Scheffer GL, Maliepaard M, Pijnenborg AC, van Gastelen MA, de Jong MC, et al (2000) Breast cancer resistance protein is localized at the plasma membrane in mitoxantrone- and topotecan-resistant cell lines. Cancer Res 60: 2589–2593. 10825126

[42] Diestra JE, Scheffer GL, Catala I, Maliepaard M, Schellens JH, et al (2002) Frequent expression of the multi-drug resistance-associated protein BCRP/MXR/ABCP/ABCG2 in human tumours detected by the BXP-21 monoclonal antibody in paraffin-embedded material. J Pathol 198: 213–219. 12237881 10.1002/path.1203

[43] Caride AJ, Filoteo AG, Enyedi A, Verma AK, Penniston JT (1996) Detection of isoform 4 of the plasma membrane calcium pump in human tissues by using isoform-specific monoclonal antibodies. Biochem J 316 (Pt 1): 353–359. 10.1042/bj3160353 PMC1217347 8645230

[44] Hegedus C, Ozvegy-Laczka C, Apati A, Magocsi M, Nemet K, et al (2009) Interaction of nilotinib, dasatinib and bosutinib with ABCB1 and ABCG2: implications for altered anti-cancer effects and pharmacological properties. Br J Pharmacol 158: 1153–1164. 19785662 10.1111/j.1476-5381.2009.00383.x PMC2785536

[45] Fischer S, Lakatos PL, Lakatos L, Kovacs A, Molnar T, et al (2007) ATP-binding cassette transporter ABCG2 (BCRP) and ABCB1 (MDR1) variants are not associated with disease susceptibility, disease phenotype response to medical therapy or need for surgery in Hungarian patients with inflammatory bowel diseases. Scand J Gastroenterol 42: 726–733. 17505995 10.1080/00365520601101559

[46] Ozvegy-Laczka C, Laczko R, Hegedus C, Litman T, Varady G, et al (2008) Interaction with the 5D3 monoclonal antibody is regulated by intramolecular rearrangements but not by covalent dimer formation of the human ABCG2 multidrug transporter. J Biol Chem 283: 26059–26070. 18644784 10.1074/jbc.M803230200 PMC3258862

[47] Cusatis G, Sparreboom A (2008) Pharmacogenomic importance of ABCG2. Pharmacogenomics 9: 1005–1009. 18681776 10.2217/14622416.9.8.1005

[48] Kobayashi D, Ieiri I, Hirota T, Takane H, Maegawa S, et al (2005) Functional assessment of ABCG2 (BCRP) gene polymorphisms to protein expression in human placenta. Drug Metab Dispos 33: 94–101. 15475413 10.1124/dmd.104.001628

[49] Sissung TM, Baum CE, Kirkland CT, Gao R, Gardner ER, et al (2010) Pharmacogenetics of membrane transporters: an update on current approaches. Mol Biotechnol 44: 152–167. 19950006 10.1007/s12033-009-9220-6 PMC6362991

[50] Li N, Palandra J, Nemirovskiy OV, Lai Y (2009) LC-MS/MS mediated absolute quantification and comparison of bile salt export pump and breast cancer resistance protein in livers and hepatocytes across species. Anal Chem 81: 2251–2259. 19209887 10.1021/ac8024009

