# Supplementary figures and images for: Expression Levels of the ABCG2 Multidrug Transporter in Human Erythrocytes Correspond to Pharmacologically Relevant Genetic Variations
Source: PLoS One. 2012 Nov 15;7(11):e48423. doi: 10.1371/journal.pone.0048423 (PMC3499528; doi:10.1371/journal.pone.0048423)

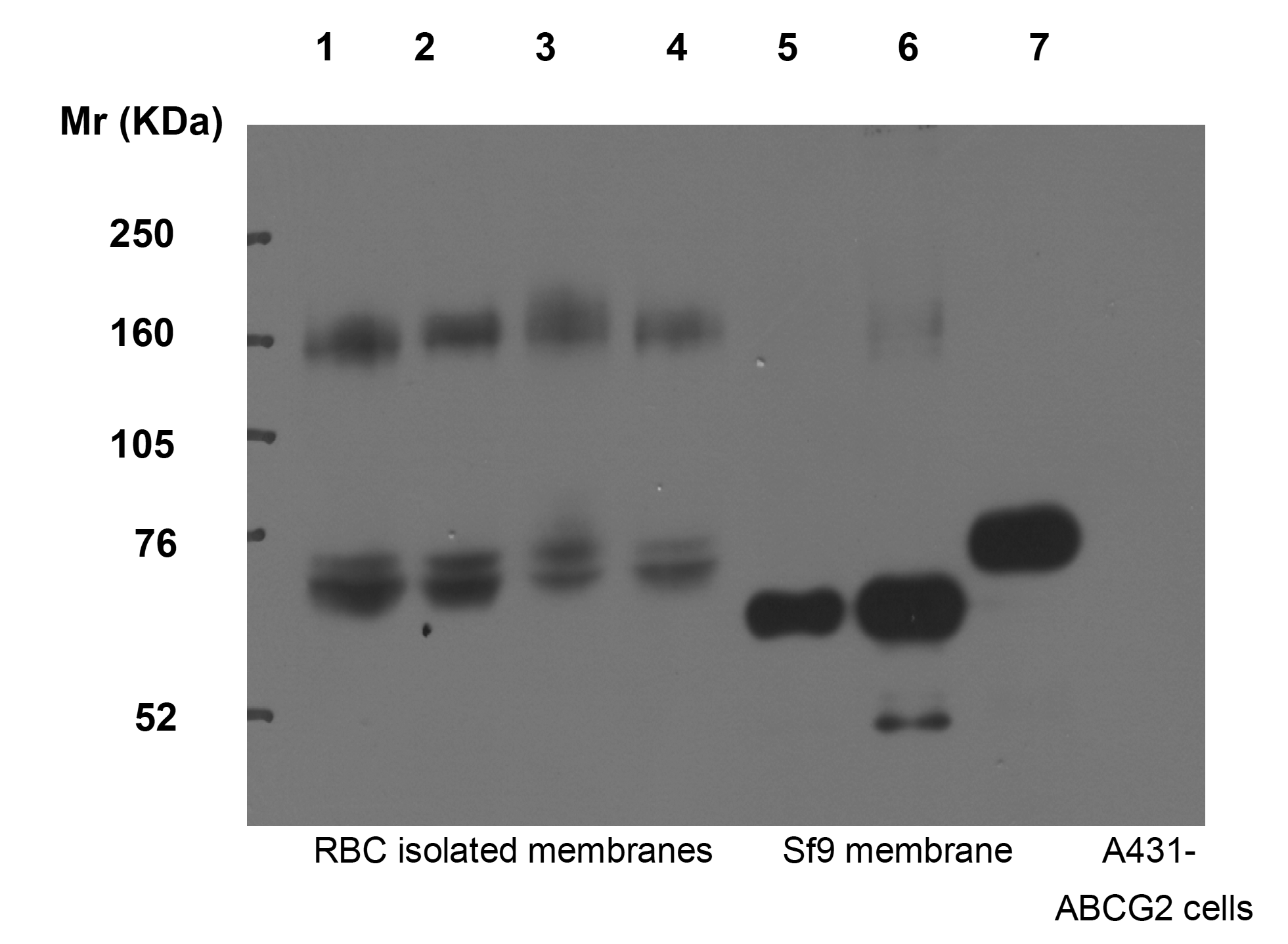

Supplement: Figure S1 — Western blot analysis of isolated red cell membrane preparations, compared to ABCG2-expressing Sf9 cell membrane preparations or A431 tumor cells, expressing ABCG2 [1] . (TIF) [file pone.0048423.s001.tif]

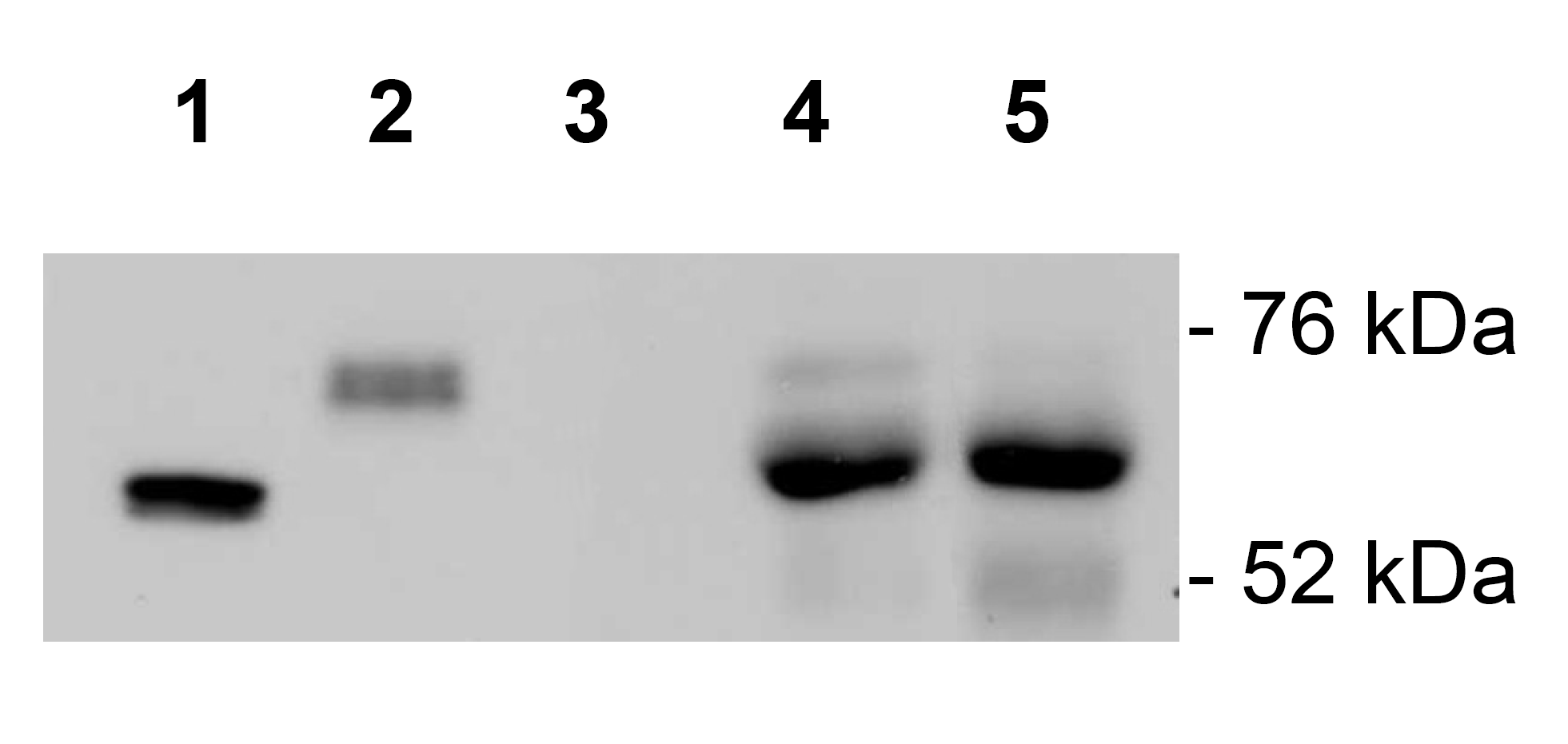

Supplement: Figure S2 — Comparison of ABCG2 expression on Western blot – detection by BXP21 antibody. (TIF) [file pone.0048423.s002.tif]

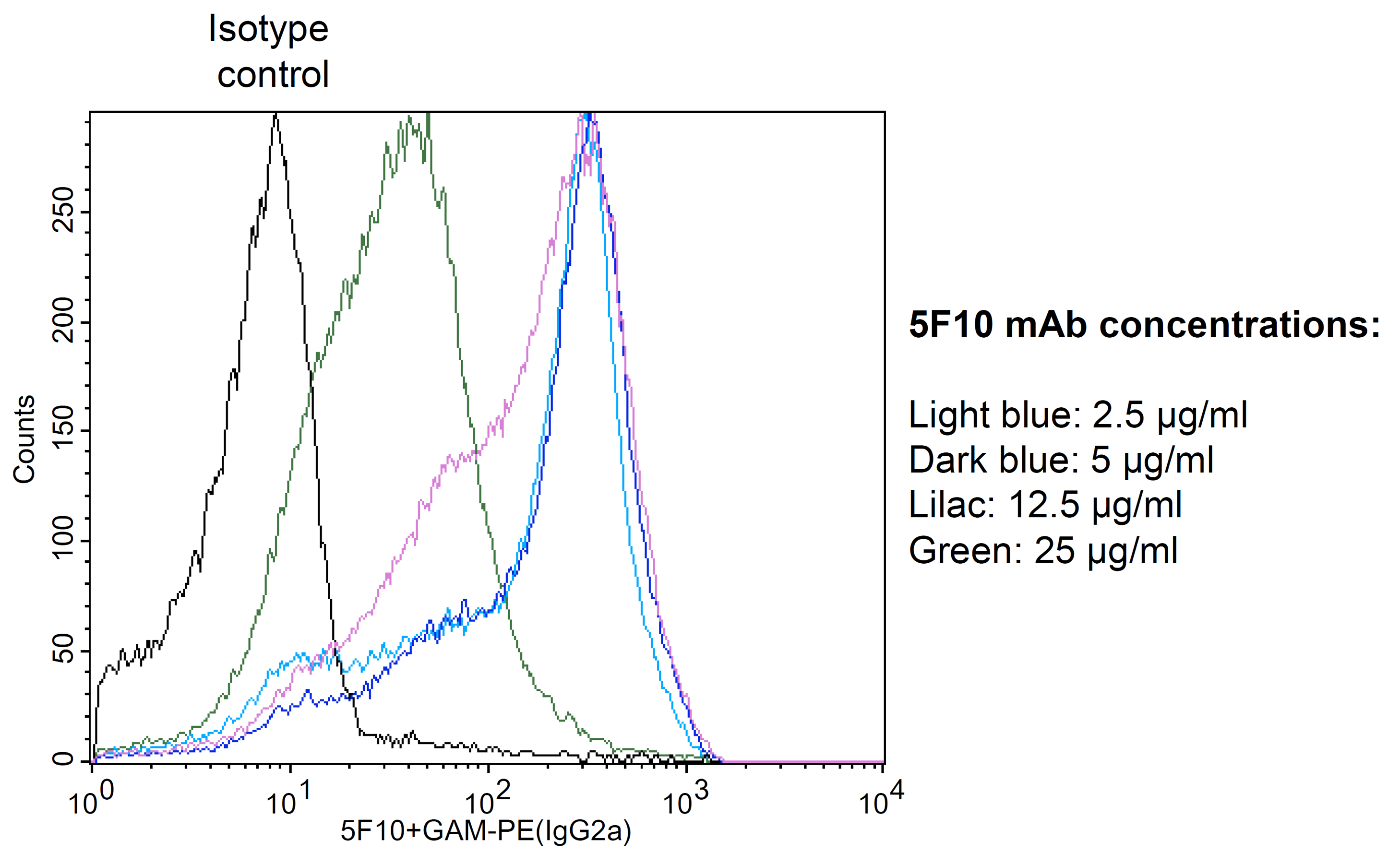

Supplement: Figure S3 — Calibration of 5F10 antibody binding and saturation. (TIF) [file pone.0048423.s003.tif]
