# Supplementary material for: Expression Levels of the ABCG2 Multidrug Transporter in Human Erythrocytes Correspond to Pharmacologically Relevant Genetic Variations
Source: PLoS One. 2012 Nov 15;7(11):e48423. doi: 10.1371/journal.pone.0048423 (PMC3499528; doi:10.1371/journal.pone.0048423)
Supplement: Materials S1 — (DOC) [file pone.0048423.s004.doc]

**Supplementary Materials**

**Kasza et al., Expression levels of the ABCG2 multidrug transporter in human erythrocytes correspond to pharmacologically relevant genetic variations.**

**1. Western blot experiments**

**1.A.** In order to document the expression of ABCG2 in the red cell membrane and compare the expression levels to those in known expression systems, we have performed numerous Western blot experiments by using isolated red cell membranes, isolated Sf9 insect cell membranes expressing the human ABCG2 protein, and A431 cells overexpressing ABCG2 [1,2] As shown in Fig. S1, in accordance with previous data in the literature [3,4,5]. we detected both the monomeric and dimeric forms of ABCG2 in the red cell membrane preparations. Based on several similar blots and comparing expression in the Sf9 membranes (corresponding to about 4% of total membrane proteins), and expression in the red cell membranes, we calculated an expression between 1-2,000 copies of ABCG2 in normal human erythrocytes.

**Figure S1**. Western blot analysis of isolated red cell membrane preparations, compared to ABCG2-expressing Sf9 cell membrane preparations or A431 tumor cells, expressing ABCG2 [1].

Lanes 1-4: Red cell membrane preparations from different donors, 10 µg /lane,

Lanes 5-6: Sf9 cell membrane, expressing human ABCG2, 0.1 and 0.3 µg,

Lane 7: A431 tumor cells expressing human ABCG2, 10 µg.

**
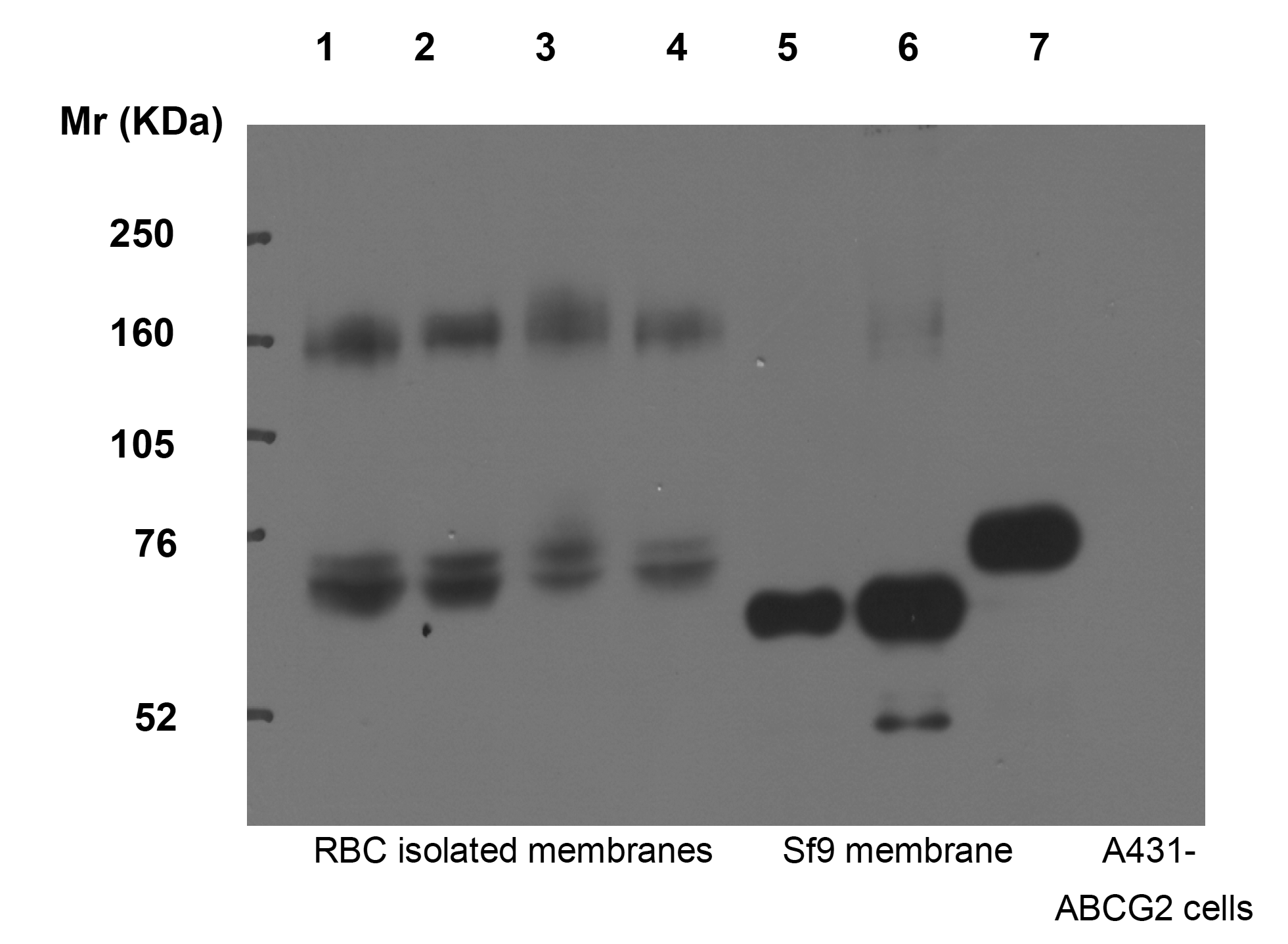
**

**1.B.** In order to compare the relative expression of ABCG2 in the various donor red cells we performed Western blot experiments by using intact red cells from donors with a heterozygous stop mutation, compared to wild-type donors.

**Figure S2. Comparison of ABCG2 expression on Western blot – detection by BXP21 antibody.**

We compared the expression levels of the ABCG2 protein in intact red cells to that in isolated membranes of Sf9 cells expressing the human ABCG2 protein (lane 1), K562 human erythroleukemia cells (lane 3, control) and K562 cells expressing ABCG2 based on lentiviral transduction (lane 2, K562-ABCG2). Total red cell preparations (15 µl of blood) were dissolved in 85 µl special disaggregation buffer, containing SDS and urea. Samples were applied from freshly drown blood of a donor homozygous for wt *ABCG2* (lane 4), and of a donor heterozygous for an *ABCG2* stop mutation (lane 5).

Note: When using an SDS+urea disaggreagation buffer and fresh red cells, only the monomeric form of ABCG2 is detected. The ABCG2 protein expressed in Sf9 cells is under-glycosylated, a high level glycosylation is observed in the K562 cells, while an intermediate level of glycosylation is seen in the red blood cells.


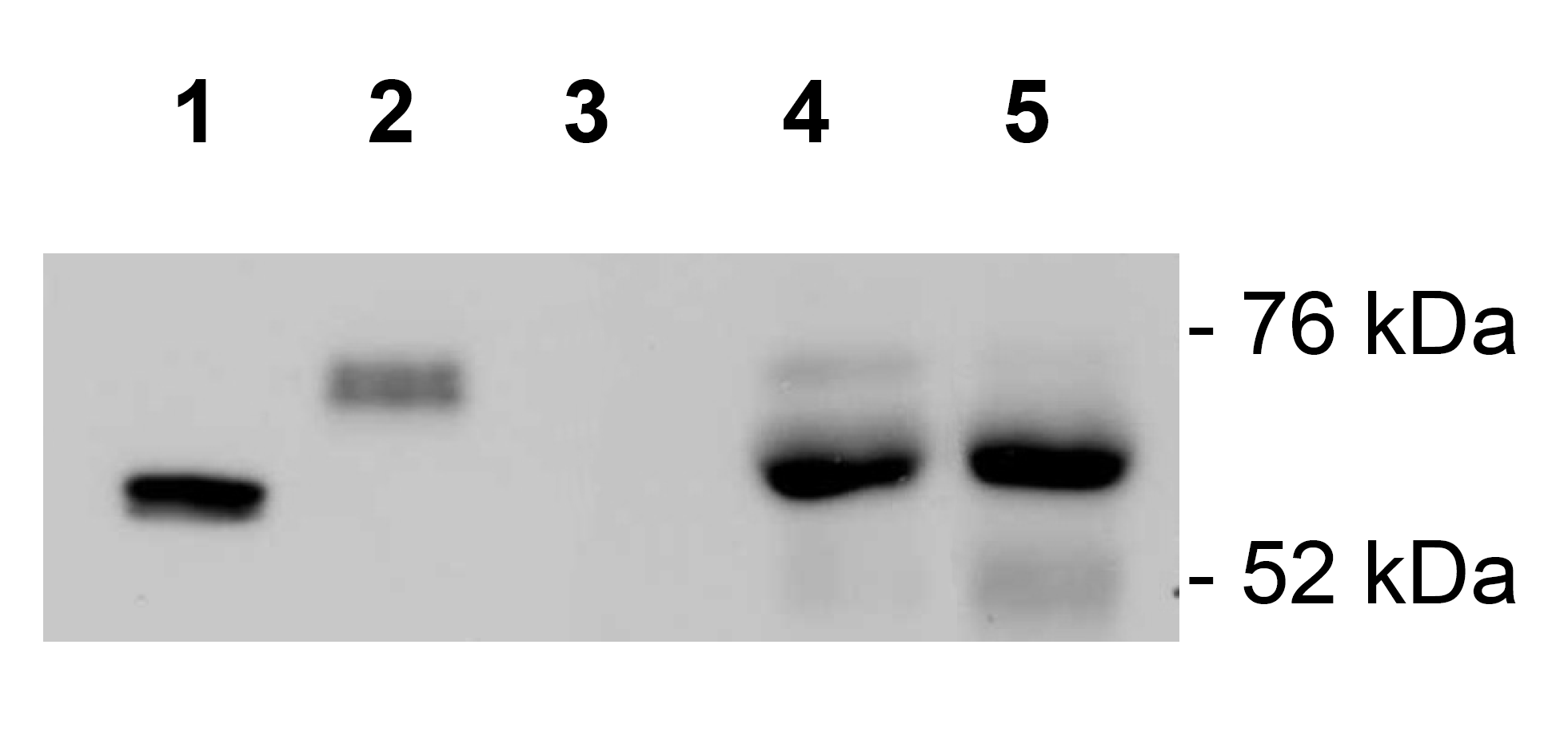


Lane 1: Sf9 cell-ABCG2, 0.03 µg membrane protein,

Lane 2: K562-ABCG2 cells, 5 µg total protein,

Lane 3: K562 control cells, 5 µg total protein,

Lane 4: Donor red cells (wt ABCG2), 50 µg total protein,

Lane 5: Donor red cells (heterozygous stop ABCG2), 50 µg total protein.

As documented, the ABCG2 bands are well visible, but there is no appreciable difference in the ABCG2 bands of these individuals by this technology (as reinforced by several similar blotting experiments). These experiments clearly point that this technology, while laborious and time consuming, cannot be used to evaluate small differences in the expression levels between various blood samples.

**2. Analysis of intra- and inter-assay variations**

A detailed analysis of the intra- and inter-assay variations obtained in this study (see Results) is presented in Table S1.

**Table S1. Characteristics of the assay performances described by intra- and interassay coefficients of variation (CV%).**

|  | **Intraassay CV%** | **Interassay CV%** |
| --- | --- | --- |
| 5D3 whole cell | 3.8% | 7.1% |
| 5D3 ghost | 7.8% | 17.9% |
| BXP21 | 6.2% | 16.6% |
| BXP34 | 11.2% | 12.4% |
| **ABCG2 factor** | **5.3%** | **10.1%** |

**3. Calibration of antibody labeling**

Here we show the calibration of the labeling of the human red cell membrane PMCA (plasma membrane calcium pump) with 5F10 monoclonal antibody. We have performed similar calibrations for all the antibodies used in this study. In order to obtain maximum labeling various concentrations of the antibody were applied.

**Figure S3. Calibration of 5F10 antibody binding and saturation.**

Human RBC were fixed and the PMCA protein was quantitated in the ghost fraction by the 5F10 monoclonal antibody, recognizing all PMCA isoforms (see Methods section). Maximum labeling was obtained by 2.5 - 5 µg/ml antibody concentration, higher mAb concentrations reduced specific binding.

**
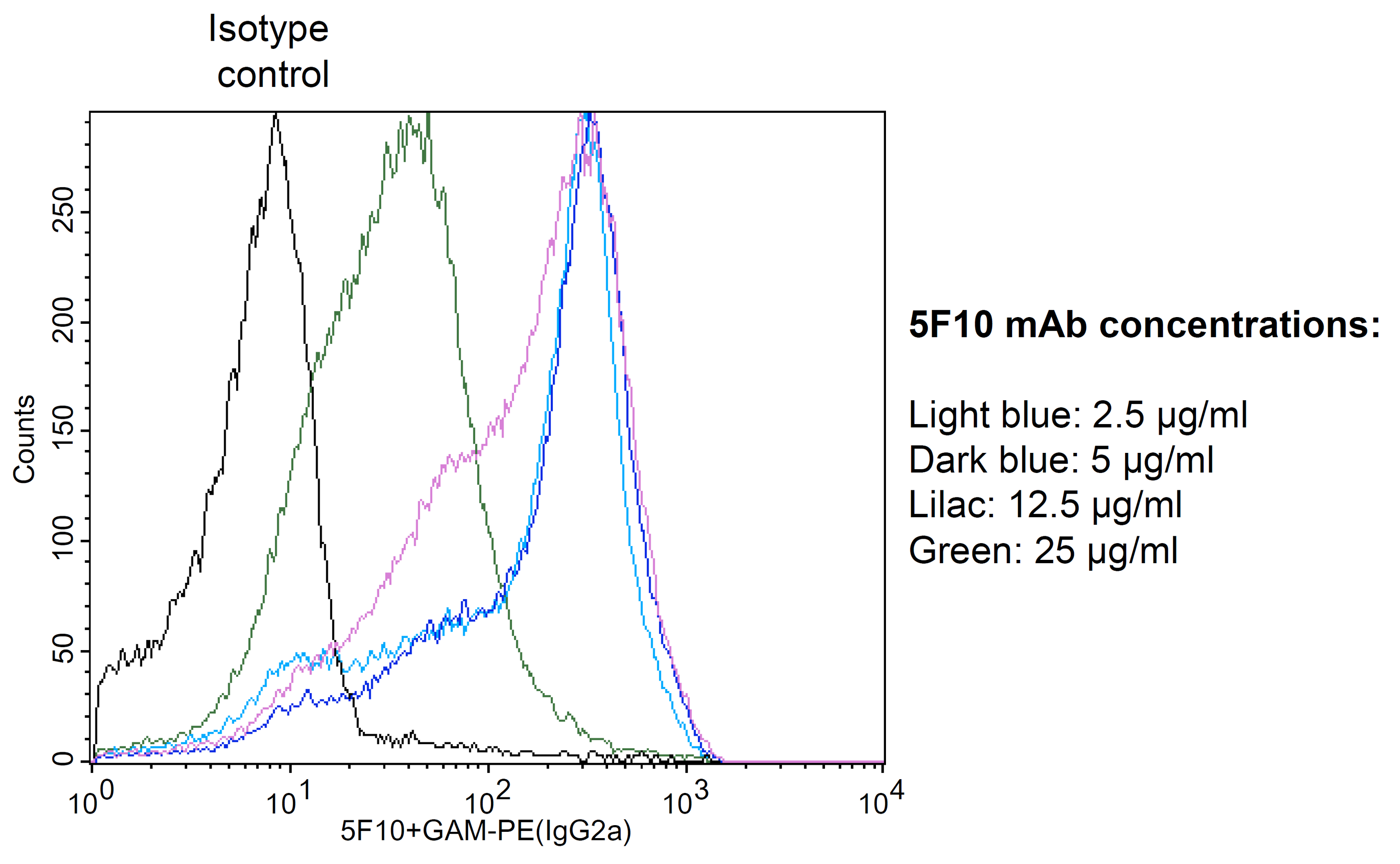
**

**References**

1. Ozvegy C, Varadi A, Sarkadi B (2002) Characterization of drug transport, ATP hydrolysis, and nucleotide trapping by the human ABCG2 multidrug transporter. Modulation of substrate specificity by a point mutation. J Biol Chem 277: 47980-47990.

2. Telbisz A, Muller M, Ozvegy-Laczka C, Homolya L, Szente L, et al. (2007) Membrane cholesterol selectively modulates the activity of the human ABCG2 multidrug transporter. Biochim Biophys Acta 1768: 2698-2713.

3. de Wolf CJ, Yamaguchi H, van der Heijden I, Wielinga PR, Hundscheid SL, et al. (2007) cGMP transport by vesicles from human and mouse erythrocytes. Febs J 274: 439-450.

4. Leimanis ML, Georges E (2007) ABCG2 membrane transporter in mature human erythrocytes is exclusively homodimer. Biochem Biophys Res Commun 354: 345-350.

5. Saison C, Helias V, Ballif BA, Peyrard T, Puy H, et al. (2012) Null alleles of ABCG2 encoding the breast cancer resistance protein define the new blood group system Junior. Nat Genet 44: 174-177.
